# Isomerization of Perylene Diimide Based Acceptors Enabling High‐Performance Nonfullerene Organic Solar Cells with Excellent Fill Factor

**DOI:** 10.1002/advs.201802065

**Published:** 2019-01-30

**Authors:** Zhenghui Luo, Tao Liu, Zhanxiang Chen, Yiqun Xiao, Guangye Zhang, Lijun Huo, Cheng Zhong, Xinhui Lu, He Yan, Yanming Sun, Chuluo Yang

**Affiliations:** ^1^ Hubei Key Lab on Organic and Polymeric Optoelectronic Materials Department of Chemistry Wuhan University Wuhan 430072 P. R. China; ^2^ Shenzhen Key Laboratory of Polymer Science and Technology College of Materials Science and Engineering Shenzhen University Shenzhen 518060 P. R. China; ^3^ School of Chemistry Beihang University Beijing 100191 P. R. China; ^4^ Department of Chemistry and Hong Kong Branch of Chinese National Engineering Research Center for Tissue Restoration & Reconstruction Hong Kong University of Science and Technology (HKUST) Clear Water Bay Kowloon Hong Kong 999077 P. R. China; ^5^ Department of Physics Chinese University of Hong Kong New Territories Hong Kong 999077 P. R. China

**Keywords:** fill factor, isomerization, organic solar cells, perylene diimide, small molecular acceptors

## Abstract

A strategy that employs the central‐core regiochemistry to develop two isomeric perylene diimide (PDI)‐based small molecular acceptors (SMAs), BPT‐Se and BPT‐Se1, is introduced, and the effect of the central‐core regiochemistry on the optical, electronic, charge‐transport, photovoltaic, and morphological properties of the molecules and their devices is investigated. The PDBT‐T1:BPT‐Se1‐based device delivers a power conversion efficiency (PCE) of 9.54% with an excellent fill factor (FF) of 73.2%, while the BPT‐Se‐based device yields a PCE of 7.78%. The large improvement of PCE upon isomerization of BPT‐Se should be ascribed to the concurrent enhancement of FF, short circuit current ( *J*_SC_), and open circuit voltage (*V*_OC_) of the PDBT‐T1:BPT‐Se1 devices. The higher FF of the organic solar cells (OSCs) based on PDBT‐T1:BPT‐Se1 can be attributed to the higher charge dissociation and charge collection efficiency, less bimolecular combination, more balanced *µ*_h_/*µ*_e_, better molecular packing and a more favorable morphology. It is worth mentioning that the FF of 73.2% is the highest value for PDI‐based SMAs OSCs to date. The result shows that regiochemistry of the central core in PDI‐based SMAs greatly affects the physicochemical properties and photovoltaic performance. The success of the isomerization strategy offers exciting prospects for the molecular design of PDI‐based SMAs.

As one of the hottest research priorities at present, fullerene‐free organic solar cells (OSCs) have attracted much attention.^1^, ^2^, ^3^, ^4^, ^5^, ^6^, ^7^, ^8^, ^9^ In comparison with fullerene acceptors, such as PC_61_BM ([6,6]‐phenyl‐C_61_‐butyric acid methyl ester) and ICBA (indene‐C_60_ bis‐adduct), nonfullerene small molecular acceptors (SMAs) outperform fullerene derivatives in terms of absorption, energy level tunability, morphological stability, and purification cost.^10^, ^11^, ^12^, ^13^, ^14^, ^15^, ^16^, ^17^, ^18^, ^19^ Significant progress in SMAs‐based OSCs has been made in the past few years.^20^, ^21^, ^22^, ^23^, ^24^, ^25^ The device based on SMAs achieved power conversion efficiencies (PCEs) over 13%, which surpassed the performance of PC_61_BM/PC_71_BM‐based devices.^26^, ^27^, ^28^, ^29^ Among these SMAs, fused‐ring electron acceptors (FREAs) and perylene diimide (PDI) derivatives have been extensively studied and are considered as the SMAs with the highest potential to replace fullerenes.^30^, ^31^, ^32^, ^33^, ^34^, ^35^, ^36^, ^37^, ^38^, ^39^, ^40^

PDI derivatives possess excellent electron mobility, strong electron affinity, outstanding chemical/thermal robustness, high absorption coefficients, and tunable absorption ranges.^41^, ^42^, ^43^ Despite of these merits, early generations of PDI derivatives show large conjugated planar structures, whose excessively strong aggregation and oversized domain size impede the development of device performance.^44^, ^45^, ^46^, ^47^, ^48^, ^49^ Since then, several strategies have been introduced to suppress the excessive aggregation propensity, including constructing twisted PDI dimers (or trimers) through connecting the α, β positions or the N‐position,^43^, ^44^, ^45^, ^50^, ^51^, ^52^ and linking PDI units through different functional groups such as triphenylamine,^53^, ^54^ tetraphenyl ethylene,^35^ and spirofluorene.^51^, ^55^ In order to further improve the efficiency of PDI‐based device, constructing fused‐ring PDI acceptors or changing the connection point of functional groups (or PDI) are currently the main strategies to optimize the structure of PDI‐based SMAs.^56^, ^57^, ^58^, ^59^

On the other hand, regiochemistry of the central units can also play an important role in the determining the characteristics of a molecule. This concept has intrigued growing research interest in the design of FREAs and has been proven to influence the electronic structure, absorption spectra, charge transport and molecular stacking, thus leading to different device performance.^60^, ^61^, ^62^, ^63^ For example, Zhan and coworkers developed two isomeric FREAs, FNIC1 and FNIC2. FNIC2 exhibits red‐shifted absorption spectra (42 nm) relative to that of FNIC1, which leads to the significantly enhanced *J*_SC_, thus a higher PCE.^61^ Li and co‐workers reported a side‐chain isomerization on ITIC by replacing 4‐hexylphenyl with 3‐hexylphenyl, the isomeric counterpart m‐ITIC‐based PSCs showed obviously enhanced PCEs due to the higher electron mobility and improved π–π stacking of m‐ITIC.^64^ From a design point of view, the regiochemistry of the central units could be an effective route for constructing efficient PDI‐based acceptors as well. Meanwhile, employing this strategy in PDI‐based SMAs could offer a new understanding on the structure–property relationship that is beneficial for enhancing device performance of nonfullerene OSCs. Nevertheless, this strategy has not yet been investigated in PDI‐based SMAs.

In this work, we report two isomeric PDI‐based acceptors with Se‐annulated PDIs as peripheral groups, namely, BPT‐Se and BPT‐Se1 (Scheme 1). The central core of BPT‐Se is 4,8‐di(thiophen‐2‐yl)benzo[1,2‐*b*:4,5‐*b*']dithiophene (BDT‐Th), which is a continuation of our previous work,^41^ while that of BPT‐Se1 is 4,5‐di(thiophen‐2‐yl)benzo[1,2‐*b*:6,5‐*b*']dithiophene (*i*‐BDT‐Th). BPT‐Se1 displays a stronger absorption, a shallower LUMO energy level, a less twisted intramolecular geometry and a higher electron mobility relative to those of BPT‐Se. When made into photovoltaic devices, the BPT‐Se1‐based device gave a higher PCE of 9.54% with an outstanding fill factor (FF) of 0.732 compared with the reference BPT‐Se‐based device (PCE = 7.78%). The performance of BPT‐Se1 is among the best results for PDI‐based OSCs. Along with the device characterization, grazing incident X‐ray wide‐angle scattering (GIWAXS), and atomic force microscopy (AFM) studies explained the device performance trend and are consistent with our structural design expectation. These results indicate that isomerization can serve as another important route to regulate the molecular properties of the PDI‐based acceptors and thus improve the photovoltaic performance of PDI‐based nonfullerene OSCs.

**Scheme 1:**
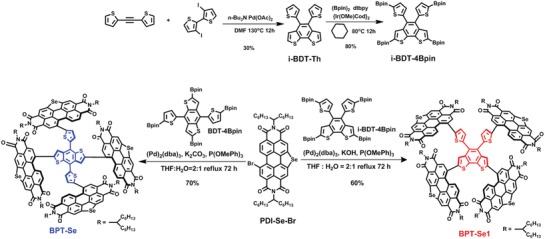
Synthesis of BPT‐Se and BPT‐Se1.

The synthetic routes to BPT‐Se and BPT‐Se1 are illustrated in Scheme 1 and Scheme S1 in the Supporting Information. Palladium‐catalyzed coupling between 1,2‐di(thiophen‐2‐yl)ethyne and 3,3'‐diiodo‐2,2'‐bithiophene generated the core *i*‐BDT‐Th. Next, selective borylation of *i*‐BDT‐Th provided the product of *i*‐BDT‐4Bpin. Thereafter, Suzuki coupling between Se‐annulated PDI and BDT‐4Bpin and *i*‐BDT‐4Bpin yielded BPT‐Se and BPT‐Se1, respectively. Both SMAs were fully characterized by ^1^H NMR, ^13^C NMR, mass spectrometry, and elemental analysis. They showed good solubility in common organic solvents, including chlorobenzene and chloroform.

As shown in **Figure** **1**a, the ultraviolet visible (UV–vis) absorption spectra of BPT‐Se1 exhibits two vibronic peaks between 400 and 600 nm in dilute chloroform solution. The corresponding maximum extinction coefficients (ε_max_) are 2.04 × 10^5^ m ^−1^ cm^−1^ at 476 nm and 2.05 × 10^5^ m ^−1^ cm^−1^ at 511 nm, which are significantly higher than those of BPT‐Se (1.69 × 10^5^ m ^−1^ cm^−1^ at 476 nm and 1.85 × 10^5^ m ^−1^ cm^−1^ at 510 nm). BPT‐Se and BPT‐Se1 in thin film show similar absorption spectra to their solution ones, indicating that the intermolecular aggregation is well suppressed. Meanwhile, BPT‐Se1 displays a slightly larger optical bandgap (2.07 eV) than BPT‐Se (2.04 eV). The absorption profiles of these SMAs are complementary to the strong absorption of PDBT‐T1, the highly efficient polymer donor we reported previously that absorbs strongly in the range of 500–700 nm.^65^ As shown in Figure S1 in the Supporting Information, the photoluminescence (PL) of both BPT‐Se and BPT‐Se1 are highly quenched by the donor PDBT‐T1, indicative of efficient charge transfer between PDBT‐T1 and the acceptors.

**Figure 1:**
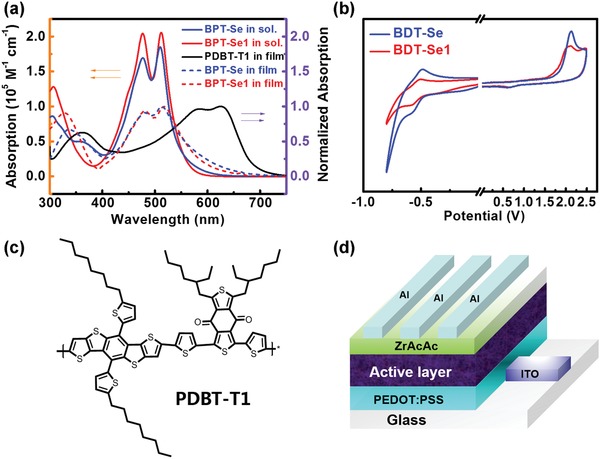
a) Absorption spectra of BPT‐Se and BPT‐Se1 in solution, and normalized absorption spectra of BPT, BPT‐S and PDBT‐T1 in film; b) cyclic voltammograms of BPT‐Se and BPT‐Se1; c) chemical structure of the donor PDBT‐T1; d) device configuration of the studied OSCs.

Regarding the electrochemical characteristics, the energy levels of BPT and BPT‐Se were investigated by cyclic voltammetry (CV). The lowest unoccupied molecular orbital (LUMO)/highest occupied molecular orbital (HOMO) levels of BPT‐Se and BPT‐Se1 estimated from the onsets of reduction/oxidation potentials are −3.85/−5.92 and −3.81/−5.85 eV, respectively. The shallower LUMO energy level for BPT‐Se1 contributes to the higher open circuit voltage (*V*_OC_) relative to that of BPT‐Se (see later section). Also, we employed ultraviolet photoelectron spectroscopy to confirm the energy levels obtained from CV measurements (Figure S2, Supporting Information). The ionization potentials of BPT‐Se and BPT‐Se1 were calculated to be 6.06 and 5.96 eV, respectively. The calculated LUMO energy levels from the ionization potential and optical bandgap are −4.02 and −3.89 eV for BPT‐Se and BPT‐Se1, respectively, which verifies the result that from BPT‐Se to BPT‐Se1, the LUMO increases. Furthermore, the calculated LUMO energy levels from density functional theory by using Gaussian at the B3LYP‐D3(BJ)/def2‐SVP level are −3.68 eV for BPT‐Se and −3.55 eV for BPT‐Se1 (Figure S3, Supporting Information), which are consistent with the CV measurement. The distribution of HOMO and LUMO orbitals are shown in Figure S3 in the Supporting Information. In BPT‐Se, the LUMO and HOMO electron density mainly localize in the PDI unit and BDT‐Th core, respectively. However, in BPT‐Se1, the LUMO orbitals concentrate in PDI unit while the HOMO orbitals are distributed on PDI‐BDT‐PDI. In addition, the optimized molecular geometries of BPT‐Se and BPT‐Se1 are presented in Figure S4 in the Supporting Information. In BPT‐Se, the dihedral angles between PDIs and thiophene, PDIs and BDT, are 76.0°, 76.0°, 70°, and 67°, respectively, while in BPT‐Se1, the dihedral angles between PDIs and thiophene, PDIs and BDT, are 69°, 67°, 69°, and 64°, respectively. The slightly less twisted molecular geometry for BPT‐Se1 could be beneficial for improving the intermolecular packing relative to that of BPT‐Se, which contributes to enhancing the charge transport ability, thus obtain the larger PCE in BPT‐Se1‐based device (as discussed in the device characterization section). Consequently, the electron mobilities of BPT‐Se and BPT‐Se1 were calculated to be 4.63 × 10^−4^ and 5.82 × 10^−4^ cm^2^ V^−1^ s^−1^ by utilizing the space charge‐limited current (SCLC) method (Figure S5, Supporting Information), respectively. The higher electron mobility of BPT‐Se1 could be attributed to the improved intermolecular packing in BPT‐Se1.

In order to evaluate the potential application of these two compounds, OSCs were fabricated with a conventional architecture of indium tin oxide/PEDOT:PSS (poly(3,4‐ethylenedioxythiophene):poly(styrene‐sulfonate))/PDBT‐T1:acceptors/ZrAcAc (zirconium acetylacetonate)/Al. We optimized the thermal annealing temperature of the PDBT‐T1:BPT‐Se and PDBT‐T1:BPT‐Se1 from 90 to 110 °C, and blend ratios of the PDBT‐T1:BPT‐Se and PDBT‐T1:BPT‐Se1 from 1.5:1 to 1:1.5. The corresponding data are summarized in Tables S1 and S2 in the Supporting Information. The optimal donor/acceptor weight ratio is 1:1 with a total concentration of 20 mg mL^−1^ in *o*‐dichlorobenzene, and the best annealing temperature is about at 100 °C (for 5 min). The typical current density–voltage (*J*–*V*) characteristics and photovoltaic parameters are displayed in **Figure** **2** and **Table** **1**, respectively. The optimized OSCs based on PDBT‐T1:BPT‐Se show a PCE of 7.78% with a *V*_OC_ of 1.039 V, a short‐circuit current density (*J*_SC_) of 11.10 mA cm^−2^ along with an FF of 0.675. Notably, the best PDBT‐T1:BPT‐Se1 achieved a higher PCE of 9.54% with a *V*_OC_ of 1.059 V, a *J*_SC_ of 12.30 mA cm^−2^, and an FF of 73.2%. The large improvement of PCEs upon isomerization of BPT‐Se should be ascribed to the overall enhancement of FF, *J*_SC_, and *V*_OC_ in the PDBT‐T1:BPT‐Se1 devices. The larger *V*_OC_ in BPT‐Se1‐based device is in agreement with the higher LUMO energy level of BPT‐Se1; the enhanced *J*_SC_ and FF are mainly attributed to the favorable morphology of the blend film. To the best of our knowledge, the PCE of 9.54% is among the best results for efficient OSCs based on PDI acceptors. It is worth noting that the FF of 0.732 is the highest value for PDI‐based SMAs OSCs to date (the best value covered in the literature was 0.715; see Figure 2c and Table S3, Supporting Information). The incident photon‐to‐current efficiency (IPCE) spectra of the optimized BPT‐Se1‐based device exhibited a significantly stronger photo‐response in the wavelength range of 430–700 nm, and the highest IPCE value of 72.5% was recorded at 520 nm for the BPT‐Se1‐based device. The *J*_SC_ values integrated from the IPCE curves are 10.791 and 11.995 mA cm^−2^ for the devices based on BPT‐Se and BPT‐Se1, respectively, which are slightly lower than those values from the *J*–*V* curves (within 3% mismatch).

**Figure 2:**
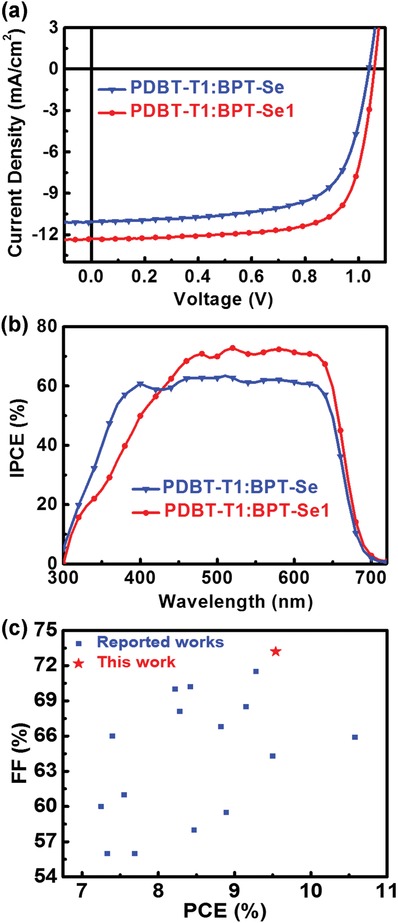
a) *J*–*V* characteristics of the best PSCs under the AM 1.5G illumination, 100 mW cm^−2^; b) EQE spectra of the corresponding devices based on PDBT‐T1:BPT‐Se and PDBT‐T1:BPT‐Se1; c) a plot of FF versus PCE for OSCs based on PDI‐based SMAs with PCEs over 7%.

**Table 1:** Photovoltaic performance and mobilities of the optimized devices based on PDBT‐T1:BPT‐Se and PDBT‐T1:BPT‐Se1

Devices	*V*_OC_ a) [V]	*J*_SC_ a) [mA cm^−2^]	FFa)	PCEa) [%]	*µ*_h_ [cm^2^ V^−1^ s^−1^]	*µ*_e_ [cm^2^ V^−1^ s^−1^]
PDBT‐T1:BPT‐Se	1.036 ± 0.006	11.05 ± 0.13	0.671 ± 0.006	7.68 ± 0.13	3.87 × 10^−4^	2.13 × 10^−4^
PDBT‐T1:BPT‐Se1	1.056 ± 0.007	12.25 ± 0.14	0.719 ± 0.010	9.29 ± 0.13	4.23 × 10^−4^	2.76 × 10^−4^

^a)^ Average values obtained from 20 devices.

Charge‐recombination mechanism of the BPT‐Se and BPT‐Se‐1‐based devices were surveyed by measuring *J*_SC_ at different light intensities (*P*_light_). The relationship between *P*_light_ and *J*_SC_ can be describe using *J*_SC_ ∝ *P*_light_^S^ (**Figure** **3**a) where *S* = 1 indicates that all the free carriers are collected and swept out before recombination, while *S* < 1 indicates a certain degree of bimolecular recombination. The *S* of the PDBT‐T1:BPT‐Se and PDBT‐T1:BPT‐Se1 devices are 0.95 and 0.97, respectively. The larger *S* value implies less bimolecular recombination in the PDBT‐T1:BPT‐Se1 blend compared with the reference of PDBT‐T1:BPT‐Se. Additionally, to understand charge collection and exciton dissociation, the relationship between photocurrent (*J*_ph_) and effective voltage (*V*_eff_) were investigated. *V*_eff_ is dependent on the internal electric field in OSCs, which affects the transport and extraction of charge carriers. As the voltage increases, the *J*_ph_ increases linearly in the low *V*_eff_ region, and then saturates at high *V*_eff_ (>2 V; Figure 3b). Saturated *J*_ph_ (*J*_sat_) of BPT‐Se1 is 12.65 mA cm^−2^, obviously larger than that of the BPT‐Se‐based device (11.50 mA cm^−2^), mainly due to the stronger light harvesting and better exciton splitting. Under short‐circuit conditions and maximal power output, the *J*_ph_/*J*_sat_ values are 0.965 and 0.795 for the PDBT‐T1:BPT‐Se blend, and 0.973 and 0.854 for the PDBT‐T1:BPT‐Se1 blend, respectively. The higher ratios of *J*_ph_/*J*_sat_ suggest that PDBT‐T1:BPT‐Se1 device has a better exciton dissociation rate and charge collection probability. To gain deeper insights into the relationship between light absorption and photocurrent generation, we calculated the maximum exciton generation rate (*G*_max_) of the two devices. *G*_max_ follows the equation, *J*_sat_ = *q* × *G*_max_ × *L*, where *L* is the thickness of blend film and *q* is the electron charge. The device of BPT‐Se1 shows a larger *G*_max_ value of 8.32 × 10^27^ m^−3^ s^−1^ relative to that of the BPT‐Se‐based device (7.56 × 10^27^ m^−3^ s^−1^), which could be attributed the stronger light absorption of BPT‐Se1. SCLC method was employed to investigate the hole (*µ*_h_) and electron mobilities (*µ*_h_) of the blend films (Figure S6, Supporting Information), and the related data are outlined in Table 1. The PDBT‐T1:BPT‐Se‐based devices display a hole mobility of 3.87 × 10^−4^ cm^2^ V^−1^ s^−1^ and an electron mobility of 2.13 × 10^−4^ cm^2^ V^−1^ s^−1^, while the hole and electron mobility of PDBT‐T1:BPT‐Se1‐based devices are calculated to be 4.23 × 10^−4^ and 2.76 × 10^−4^ cm^2^ V^−1^ s^−1^, respectively, which are higher than those of the BPT‐Se‐based device. The less bimolecular recombination, better exciton dissociation rate and charge collections probability, and more balanced hole/electron ratio are responsible for the larger FF and *J*_SC_ obtained in BPT‐Se1‐based device.

**Figure 3:**
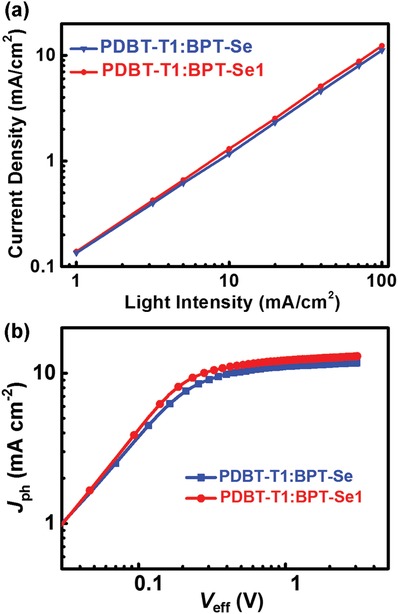
a) Light intensity dependence of *J*_SC_ of the devices based on PDBT‐T1:BPT‐Se and PDBT‐T1:BPT‐Se1; b) *J*_ph_ versus *V*_eff_ of the optimized devices based on PDBT‐T1:BPT‐Se and PDBT‐T1:BPT‐Se1.

To investigate the structure‐property relationship, morphology studies were carried out. First, AFM was utilized to survey the surface morphology of the blend film (**Figure** **4**). As can be seen from AFM height images, PDBT‐T1:BPT‐Se and PDBT‐T1:BPT‐Se1‐based blends displayed uniform and smooth surface morphologies, while BPT‐Se1‐based blend film exhibited a slightly larger root‐mean‐square surface roughness (1.06 nm), which could be ascribed to the improved molecular packing of BPT‐Se1. Clearer and more ordered nanofibrillar structures can be observed from AFM phase images for the BPT‐Se1‐based film relative to that of the BPT‐Se‐based film, which is beneficial for charge transport and extraction. Besides, GIWAXS measurements were carried out to investigate the molecular packing of the active layer.^66^ As shown in Figure S6 in the Supporting Information, pure PDBT‐T1 film shows a face‐on orientation with a lamellar‐stacking peak at *q*_r_ = 0.27 Å^−1^ and π–π stacking peak at *q*_z_ = 1.71 Å^−1^. Both BPT‐Se and BPT‐Se1 neat films exhibit ring‐like lamellar scattering at *q*_r_ = 0.27 Å^−1^ without obvious orientation preference (Figure S7, Supporting Information). Compared to BPT‐Se, BPT‐Se1 displays a relatively strong scattering intensity and a larger crystallite coherence length (CCL; BPT‐Se1 = 66.5 Å; BPT‐Se = 53.4 Å), indicating a higher crystallinity in accordance with the observed higher electron mobility. GIWAXS patterns and the corresponding intensity profiles of the PDBT‐T1:BPT‐Se and PDBT‐T1:BPT‐Se1 blend films are presented in Figure 4. Compared to the PDBT‐T1:BPT‐Se blend film, the PDBT‐T1:BPT‐Se1 blend film shows a higher crystallinity and a preferential face‐on orientation, evidenced by the stronger scattering intensity and the more prominent π–π stacking peak in the out‐of‐plane direction. The scattering peaks at *q*_r_ = 0.27 Å^−1^ for both blend films originate from the lamellar scattering of PDBT‐T1. The corresponding CCL are 93.2 Å for the PDBT‐T1:BPT‐Se blend film and 133.4 Å for the PDBT‐T1:BPT‐Se1 blend film. Furthermore, the π–π stacking peaks are at *q*_z_ = 1.66 Å^−1^ (*d* = 3.79 Å) and 1.71 Å^−1^ (*d* = 3.67 Å) for the PDBT‐T1:BPT‐Se and PDBT‐T1:BPT‐Se1 blend films, respectively. The relatively higher crystallinity and tighter π–π stacking for the PDBT‐T1:BPT‐Se1 blend film could improve charge transport, consistent with the observed higher FF and photocurrent of the devices.

**Figure 4:**
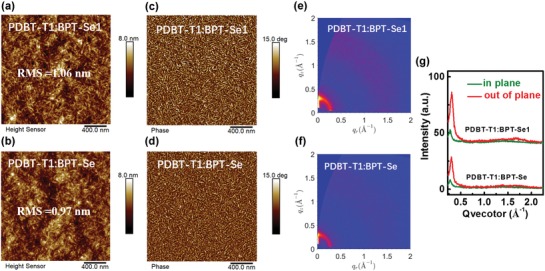
Representative AFM height images of a) PDBT‐T1:BPT‐Se1 (1:1, w/w) and b) PDBT‐T1:BPT‐Se (1:1, w/w) blend films; AFM phase images of c) PDBT‐T1:BPT‐Se1 and d) PDBT‐T1:BPT‐Se blend films; GIWAXS patterns of e) PDBT‐T1:BPT‐Se1 and f) PDBT‐T1:BPT‐Se blend films; g) corresponding intensity profiles along the in plane (green lines) and out‐of‐plane (red lines) direction of PDBT‐T1:BPT‐Se1 and PDBT‐T1:BPT‐Se blend films.

In conclusion, we designed and synthesized two isomeric PDI‐based SMAs, BPT‐Se and BPT‐Se1, and investigated the effects of the central‐core regiochemistry on optical, electronic, charge‐transport, photovoltaic, and morphological properties. GIWAXS results indicated that BPT‐Se1 has a better crystallite quality and tighter molecular packing relative to that of BPT‐Se. In addition, BPT‐Se1 exhibits a stronger absorption profile, an upshifted LUMO energy level and a higher electron mobility, which endows BPT‐Se1 with better prospects for OSC applications. Consequently, the PDBT‐T1:BPT‐Se1‐based OSC device delivers a high PCE of 9.54% with an excellent FF of 73.2%, a high *V*_OC_ of 1.059 V, and a *J*_SC_ of mA cm^−2^, while the BPT‐Se‐based device shows a PCE of 7.78%. Notably, the isomerization of BPT‐Se enhances the FF, *J*_SC_ and *V*_OC_ simultaneously and the 73.2% FF is the highest FF for the PDI‐based OSCs to date. These results demonstrate that regiochemistry of the central core in PDI‐based SMAs has a large impact on the physicochemical properties of the molecule and photovoltaic performance of the resulting device. The success of isomerization strategy thus paves the way toward high‐performance PDI‐based small molecule acceptors for nonfullerene OSCs.

## Conflict of Interest

The authors declare no conflict of interest.

## Supporting information

Supplementary
