# Supplementary material for: Isomerization of Perylene Diimide Based Acceptors Enabling High‐Performance Nonfullerene Organic Solar Cells with Excellent Fill Factor
Source: Adv Sci (Weinh). 2019 Jan 30;6(6):1802065. doi: 10.1002/advs.201802065 (PMC6425449; doi:10.1002/advs.201802065)
Supplement: Supplementary file 1 — Supplementary [file ADVS-6-1802065-s001.pdf]

Copyright WILEY-VCH Verlag GmbH & Co. KGaA, 69469 Weinheim, Germany, 2019.

ADVANCED SCIENCE
Open Access

Supporting Information

for *Adv. Sci.,* DOI: 10.1002/advs.201802065

Isomerization of Perylene Diimide Based Acceptors Enabling High-Performance Nonfullerene Organic Solar Cells with Excellent Fill Factor

Zhenghui Luo, Tao Liu, Zhanxiang Chen, Yiqun Xiao, Guangye Zhang, Lijun Huo, Cheng Zhong, Xinhui Lu,* He Yan,* Yanming Sun,* and Chuluo Yang**

Supporting Information

Isomerization of Perylene Diimide Based Acceptors Enabling High-Performance Nonfullerene Organic Solar Cells with Excellent Fill Factor

Zhenghui Luo, Tao Liu, Zhanxiang Chen, Yiqun Xiao, Guangye Zhang, Lijun Huo, Cheng Zhong, Xinhui Lu,* He Yan,* Yanming Sun* and Chuluo Yang**

Z. Luo, Z. Chen, Dr. C. Zhong, Prof. C. Yang,
Hubei Key Lab on Organic and Polymeric Optoelectronic Materials, Department of Chemistry, Wuhan University, Wuhan 430072, P. R. China,
E-mail: clyang@whu.edu.cn

Z. Luo, Prof. C. Yang
Shenzhen Key Laboratory of Polymer Science and Technology, College of Materials Science and Engineering, Shenzhen University, Shenzhen 518060, China.

Dr. T. Liu, Prof. L. Huo, Prof. Y. Sun
School of Chemistry, Beihang University, Beijing 100191, P. R. China
E-mail: sunym@buaa.edu.cn

Dr. T. Liu, Dr. G. Zhang, Prof. H.Yan
Department of Chemistry and Hong Kong Branch of Chinese National Engineering Research Center for Tissue Restoration & Reconstruction, Hong Kong University of Science and Technology (HKUST), Clear Water Bay, Kowloon, Hong Kong 999077, China
Email: liutaozhx@ust.hk; hyan@ust.hk

Y. Xiao, Prof. X. Lu
Department of Physics, Chinese University of Hong Kong, New Territories, Hong Kong 999077, China
Email: xinhui.lu@cuhk.edu.hk

Materials and Measurements

All solvents and reagents were used as received from commercial sources and used without further purification unless otherwise specified. ^1H NMR (400 MHz) and ^{13}C NMR (100 MHz) spectra were measured on a Bruker AVANCE III HD 400MHz spectrometers. Mass spectra were recorded on a Shimadzu spectrometer. UV-vis-NIR absorption spectra were recorded on a Shimadzu UV-2700 recording spectrophotometer. Cyclic voltammetry (CV) measurements were carried out on a CHI voltammetric analyzer at room temperature. Tetrabutylammonium hexafluorophosphate (n-Bu_4NPF_6, 0.1 M) was used as the supporting electrolyte. The conventional three-electrode configuration consists of a platinum working electrode with a 2 mm diameter, a platinum wire counter electrode, and a Ag/AgCl wire reference electrode. Cyclic voltammograms were obtained at a scan rate of 100 mV/s. PL spectra were measured with a Shimadzu RF-5301PC fluorescence spectrophotometer. The film morphology was measured using an atomic force microscope (AFM, Bruker-ICON2-SYS) using the tapping mode. The RMS values of the surface AFM images are averaged based on five times testing on different areas for each sample. The optimal molecular geometry was calculated using the DFT with Gaussian 09.[1] Ground state geometry optimization was performed using the B3LYP exchange-correlation functional, the def2-SVP basis set, and the density functional dispersion correction with Becke–Johnson damping [DFT-D3(BJ)].[2] Compound **1**, **2**, **5 and 6** were synthesized according to the reported literature.[3-5]

Device fabrication and characterization

The PSCs were fabricated with a structure of ITO/PEDOT: PSS /active layers/ZrAcAc/Al. A thin layer of PEDOT: PSS was deposited through spin-coating on precleaned ITO-coated glass from a PEDOT: PSS aqueous solution (Baytron P VP AI 4083 from H. C. Starck) at 4000 rpm and dried subsequently at 150 °C for 15 min in air. Then the device was transferred to a nitrogen glove box, where the active blend layer of PDBT-T1 polymer and BPT-Se (or BPT-Se1) with 0.25% DIO was spin-coated from its *o*-DCB solution onto the PEDOT: PSS layer under a spin-coating rate of 1600 rpm. After spin-coating, the active layers were annealed at 100 °C for 5 min for the devices with thermal annealing treatment. The thickness of the active layers is ca. 95 nm. The ZrAcAc as cathode buffer layer. Finally, an aluminum layer at the vacuum condition of 5×10^{-5} Pa was deposited by thermal evaporation method. All film thickness was measured by the Alpha-Step D-500 surface profilometer. The current density–voltage (*J*–*V*) characteristics of the PSCs were measured in glovebox on a computer-controlled Keithley 2450 Source-Measure Unit. Oriel Sol3A Class AAA Solar Simulator (model, Newport 94023A) with a 450 W xenon lamp and an air mass (AM) 1.5 filter was used as the light source. The light intensity was calibrated to 100 Mw cm^{-2} by a Newport Oriel 91150V reference cell. The input photon to converted current efficiency (IPCE) was measured by Solar Cell Spectral Response Measurement System QE-R3-011 (Enli Technology Co., Ltd., Taiwan). The light intensity at each wavelength was calibrated with a standard single-crystal Si photovoltaic cell. Optical microscope (Olympus BX51) was used to defined the active area (4.5 mm^2) of the device. Masks made using laser beam cutting technology to have a

well-defined area of 3.14 mm^2 were attached to define the effective area for accurate measurement. All the masked and unmasked tests gave consistent results with relative errors within 0.5%. All the device measurements were undertaken in a nitrogen glovebox.

Mobility Measurements

Hole and electron mobility were measured using the space charge limited current (SCLC) method. Device structures are ITO/MoOx/active layer/MoOx/Al for hole-only devices and ITO/ZnO/active layer/Zracac/Al for electron-only devices. The SCLC mobilities were calculated by MOTT-Gurney equation: $J = 9\varepsilon_0\varepsilon_r\mu V^2/8L^3$. Where J is the current density, ε_r is the relative dieletiric constant of active layer material usually 2-4 for organic semiconductor, herein we use a relative dielectric constant of 3, ε_0 is the permittivity of empty space, μ is the mobility of hole or electron and L is the thickness of the active layer, V is the internal voltage in the device, and $V = V_{Applied} - V_{Built\text{-}in}$ (in the hole-only and the electron-only devices, the V_{bi} values are 0.2 V and 0 V respectively), where $V_{Applied}$ is the voltage applied to the device, and $V_{Built\text{-}in}$ is the built-in voltage resulting from the relative work function difference between the two electrodes.

GIWAXS measurement

GIWAXS measurement were carried out with a Xeuss 2.0 SAXS/WAXS laboratory beamline using a Cu X-ray source (8.05 keV, 1.54 Å) and a Pilatus3R 300K detector. The incidence angle is 0.2°. The samples for GIWAXS measurements are fabricated on silicon substrates using the same recipe for the devices.

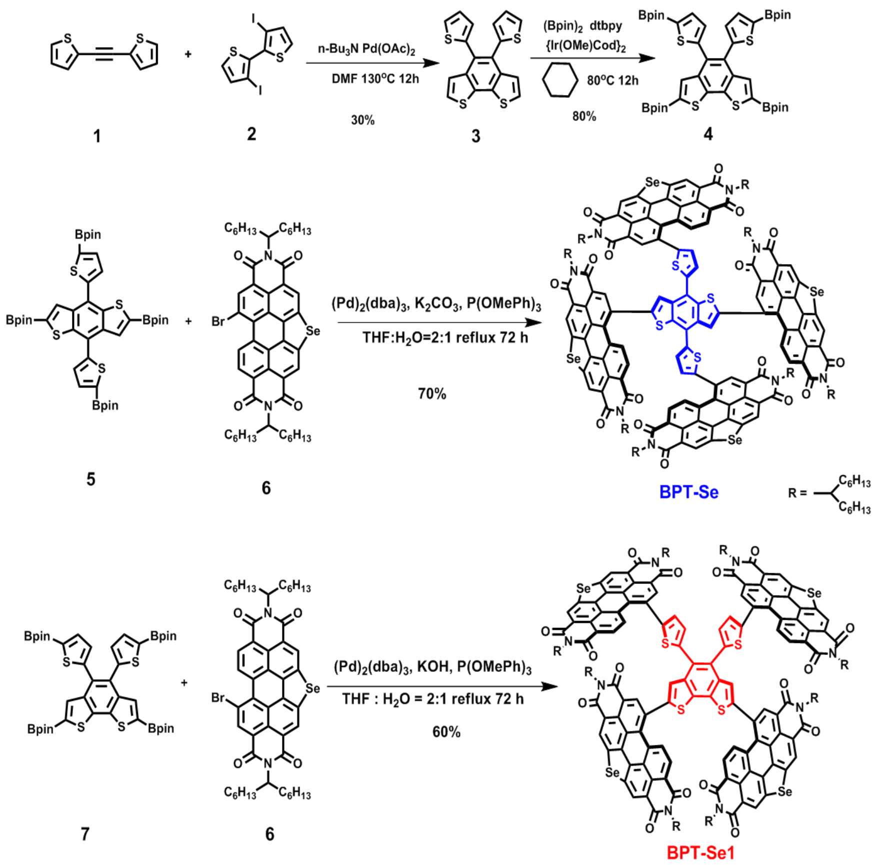

Scheme S1. Synthesis of BPT-Se, BPT-Se1.

Compound 3: In a flame-dried round bottom flask, 1 (570 mg, 3 mmol), 2 (1.25 g, 3 mmol) and NBu_3 (1 ml) were dissolved in anhydrous DMF (20 ml) and the solution was sparged with Ar. $Pd(OAc)_2$ (54 mg, 0.24 mmol) was added and the reaction mixture was stirred at 130 °C for 12 h. The mixture was cooled to r.t., diluted with CH_2Cl_2 (50 ml) and poured into H_2O (30 ml). The layers were separated and the aqueous layer was extracted with CH_2Cl_2 (3×80 ml). The combined organic layer was dried over Na_2SO_4 and the solvent was removed. The residue was purified by flash chromatography on silica

(hexanes). The title compound was isolated as a white solid (318 mg, 0.9 mmol, 30%). ^{1}H NMR ($CDCl_3$, 400 MHz): *δ* [ppm]: 7.37 (q, *J* = 5.4 Hz, 4H), 7.32 (dd, *J* = 4.6, 1.7 Hz, 2H), 7.03-7.00 (m, 4H); ^{13}C NMR (100 MHz, $CDCl_3$): *δ* [ppm]: 140.18, 137.93, 133.39, 128.76, 127.15, 126.50, 126.39, 125.67, 124.36. HRMS *m/z* calcd for $[M]^+$ ($C_{18}H_{10}S_4$), 353.9665; found, 353.9672.

Compound 4: To a mixture of compound **3** (0.445g, 1.25 mmol), $(BPin)_2$ (1.91 g, 7.52 mmol), 4,4'-ditert-butyl-2,2'-dipyridyl (91 mg, 0.34 mmol) and {Ir(OMe)Cod} (45 mg, 0.068 mmol) in 50 mL sealed tube, 20 ml anhydrous cyclohexane were added under Ar atmosphere. After reacting at 80 °C for 12 hours, the solvent was removed under reduced pressure. 0.88 g of pure compound **4** (80 %) was obtained by recrystalization in hexane and methanol. ^{1}H NMR ($CDCl_3$, 400 MHz): *δ* [ppm]:7.80 (s, 2H), 7.48 (d, *J* = 4 Hz, 2H), 6.97 (d, *J* = 4 Hz, 2H), 1.39 (s, 24H), 1.36(s, 24H). ^{13}C NMR (100 MHz, $CDCl_3$): *δ* [ppm]: 146.76, 139.39, 137.54, 137.08, 135.76, 130.51, 127.65, 84.62, 84.09, 24.84, 24.79. HRMS *m/z* calcd for $[M]^+$ ($C_{42}H_{54}B_4O_8S_4$), 858.3074; found, 858.3092.

BPT-Se: $Pd_2(dba)_3$ (16 mg, 0.017 mmol) and $P(MeOPh)_3$ (48 mg, 0.14 mmol) were added to the mixture of compound **5** (129 mg, 0.15 mmol), compound **6** (700 mg, 0.75 mmol, 5 eq), THF and 2M K_2CO_3 aqueous solution (8 mL) under Ar. The mixture was poured into methanol after refluxing 72 hours. The red precipitate was filtered and purified by column chromatography using chloroform/hexane = 2/1 (v/v) as the eluent and BPT-Se was obtained (385 mg, 0.105 mmol, yield: 70%). ^{1}H NMR ($CDCl_3$, 400

MHz): δ [ppm]: 9.53-9.22 (m, 10H), 8.94-8.75 (m, 6H), 8.60 (s, 2H), 8.35-8.27 (m, 4H), 7.93 (s, 2H), 7.43 (s, 2H), 5.37-4.61 (m, 8H), 2.38-1.95 (m, 16H), 1.92-1.68 (m, 16H), 1.52-1.00 (m, 128H), 0.88-0.52 (m, 48H); ^{13}C NMR (100 MHz, CDCl3): δ [ppm]: 165.03, 163.97, 163.37, 146.06, 143.71, 141.25, 138.37, 138.14, 137.88, 133.48, 132.34, 131.80, 128.11, 127.78, 126.21, 124.55, 124.24, 122.61, 121.29, 54.97, 53.37, 32.35, 31.68, 29.73, 29.19, 27.02, 26.93, 26.86, 23.94, 22.62, 22.52, 14.08, 14.01. HRMS (TOF) *m/z* calcd for $[M]^+$ ($C_{218}H_{242}N_8O_{16}S_4Se_4$) , 3675.3913; found, 3675.4002.

BPT-Se1: $Pd_2(dba)_3$ (16 mg, 0.017 mmol) and $P(MeOPh)_3$ (48 mg, 0.14 mmol) were added to the mixture of compound **7** (129 mg, 0.15 mmol), compound **6** (700 mg, 0.75 mmol, 5 eq), THF and 2M KOH aqueous solution (8 mL) under Ar. The mixture was poured into methanol after refluxing 72 hours. The red precipitate was filtered and purified by column chromatography using chloroform/hexane = 2/1 (v/v) as the eluent and BPT-Se1 was obtained (330 mg, 0.09mmol, yield: 60%). ^{1}H NMR ($CDCl_3$, 400 MHz): δ [ppm]: 9.49-9.27 (m, 6H), 9.25-8.25 (m, 14H), 8.05 (s, 2H), 7.71 (s, 2H), 7.48 (s, 2H), 5.30-4.50 (m, 8H), 2.39-1.98 (m, 16H), 1.92-1.70 (m, 16H), 1.52-1.00 (m, 128H), 0.90-0.50 (m, 48H); ^{13}C NMR (100 MHz, CDCl3): δ [ppm]: 165.08, 164.95, 149.77, 144.98, 143.19, 137.76, 133.65, 132.92, 131.92, 131.19, 127.95, 126.46, 126.24, 126.01,125.89, 124.56, 124.47, 124.34, 124.05, 122.93, 122.18, 54.80, 53.55, 32.32, 31.85, 29.35, 29.13, 26.99, 26.89, 26.82, 26.70, 22.59, 22.49, 14.08, 13.99. HRMS (TOF) *m/z* calcd for $[M]^+$ ($C_{218}H_{242}N_8O_{16}S_4Se_4$) , 3675.3913; found, 3675.3994.

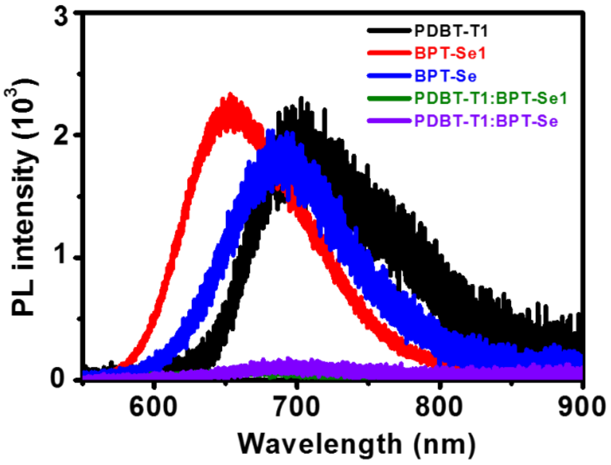

Figure S1. Photoluminescence spectra of the polymer PDBT-T1, BPT-Se and BPT-Se1 films as well as the blend films of PDBT-T1:BPT-Se (1:1, *w*/*w*) and PDBT-T1:BPT-Se1 (1:1, *w*/*w*) (excited at 514 nm).

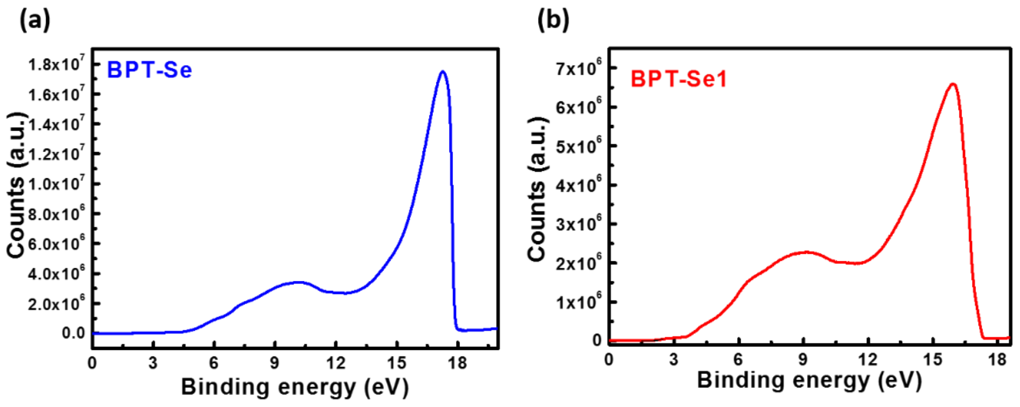

Figure S2. The ultraviolet photoelectron spectroscopy data of neat BPT-Se and BPT-Se1 films.

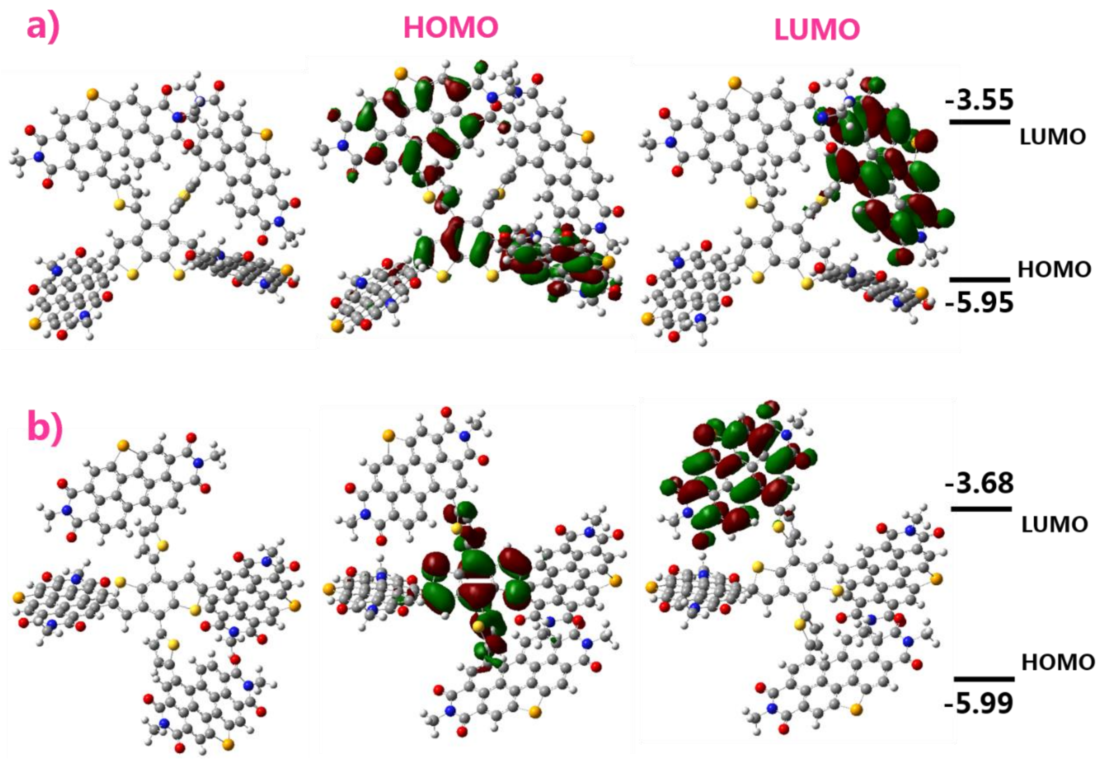

Figure S3. The chemical structures and frontier molecular orbitals obtained by DFT calculations for BPT-Se and BPT-Se1.

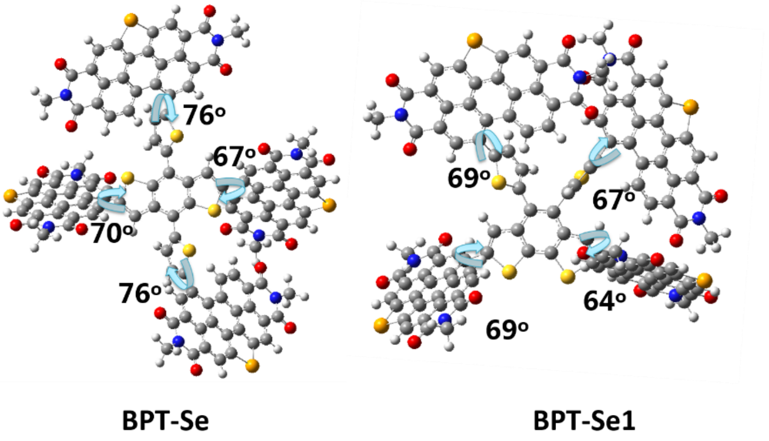

Figure S4. The optimal molecular geometries of the acceptors calculated by DFT at the B3LYP-D3(BJ)/def2-SVP level.

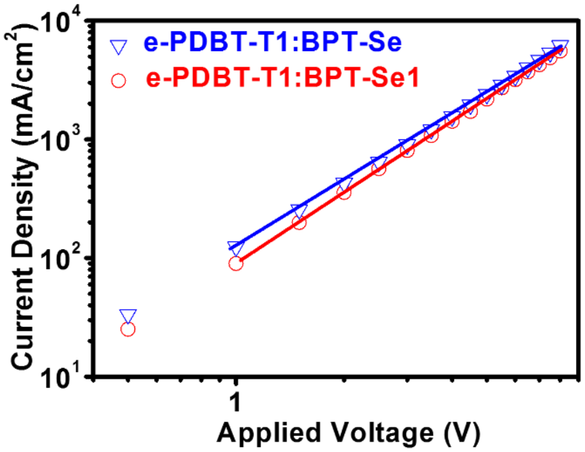

Figure S5. Current-Voltage (*J*-*V*) characteristics of BPT-Se and BPT-Se neat films in electron-only devices.

Table S1. Summarized device parameters of PDBT-T1:BPT-Se and PDBT-T1:BPT-Se1 based solar cells at the D/A ratio of 1:1 with different thermal temperature under the illumination of AM1.5G, 100 mW/cm^2.

Active Layer	Annealed T (°C)	V_{oc} (V)	J_{sc} (mA/cm^2)	FF	PCE (%)	PCE_{max} (%)
PDBT-T1: BPT-Se	RT	1.047 ± 0.005	9.19 ± 0.21	0.520 ± 0.011	4.94 ± 0.14	5.22
	90	1.036 ± 0.010	10.68 ± 0.25	0.643 ± 0.009	7.12 ± 0.22	7.44
	100	1.036 ± 0.006	11.05 ± 0.13	0.671 ± 0.006	7.68 ± 0.13	7.78
	110	1.025 ± 0.006	10.76 ± 0.25	0.666 ± 0.006	7.35 ± 0.01	7.66
PDBT-T1: BPT-Se1	RT	1.072 ± 0.004	10.09 ± 0.13	0.568 ± 0.007	6.15 ± 0.10	6.30
	90	1.060 ± 0.006	11.50 ± 0.20	0.716 ± 0.007	8.73 ± 0.23	9.04
	100	1.056 ± 0.007	12.25 ± 0.14	0.719 ± 0.010	9.29 ± 0.13	9.54
	110	1.041 ± 0.008	11.60 ± 0.21	0.720 ± 0.008	8.701 ± 0.20	9.13

Table S2. Summarized device parameters of PDBT-T1:BPT-Se and PDBT-T1:BPT-Se1 based solar cells with different D/A ratio at 100 °C for 5 min under the illumination of AM1.5G, 100 mW/cm^2.

Active Layer	Blend ratio	V_{oc} (V)	J_{sc} (mA/cm^2)	FF	PCE (%)	PCE_{max} (%)
PDBT-T1: BPT-Se	1.2:1	1.037 ± 0.005	10.23 ± 0.18	0.651 ± 0.004	6.91 ± 0.19	7.15
	1 :1	1.036 ± 0.006	11.05 ± 0.13	0.671 ± 0.006	7.68 ± 0.13	7.78
	1:1.2	1.029 ± 0.004	10.58 ± 0.21	0.643 ± 0.008	7.00 ± 0.21	7.26
PDBT-T1: BPT-Se1	1.2:1	1.058 ± 0.009	11.51 ± 0.24	0.697 ± 0.008	8.49 ± 0.24	8.86
	1 :1	1.056 ± 0.007	12.25 ± 0.14	0.719 ± 0.010	9.29 ± 0.13	9.54
	1:1.2	1.035 ± 0.007	11.80 ± 0.25	0.701 ± 0.009	8.60 ± 0.23	8.92

Table S3. The summary of PCEs over 7% in high-performance fullerene-free solar cells based on PDIs as acceptors.

Acceptor	Donor	PCE_{max}(%)	FF	V_{oc} (V)	J_{sc} (mA cm^{-1})	Ref
**BPT-Se1**	**PDBT-T1**	**9.54**	**0.732**	**1.06**	**12.30**	**This work**
BPT-S	PDBT-T1	8.28	0.681	1.02	11.94	6
SdiPBI-S	PBDTS-Se	8.22	0.700	0.91	12.90	7
SdiPBI-Se	PDBT-T1	8.42	0.702	0.96	12.49	8
TPB	PTB7-Th	8.47	0.58	0.80	18.25	9
TPH-Se	PDBT-T1	9.28	0.715	1.00	12.99	10
βTPB6-C	PTB7-T1	7.69	0.56	0.92	14.9	11
7b	P3TEA	7.55	0.61	1.13	11.03	12
Ta-PDI	PTB7-Th	9.15	0.685	0.78	17.10	13
H-tri-PDI	PBDT-TS1	7.25	0.60	0.732	16.52	14
FITP	PTB7-Th	7.33	0.56	0.99	13.24	15
SF-PDI_2	P3TEA	9.50	0.643	1.11	13.27	16
PBI-Por	PBDB-T	7.40	0.66	0.78	14.5	17
FTTB-PDI4	P3TEA	10.58	0.659	1.13	13.8	18

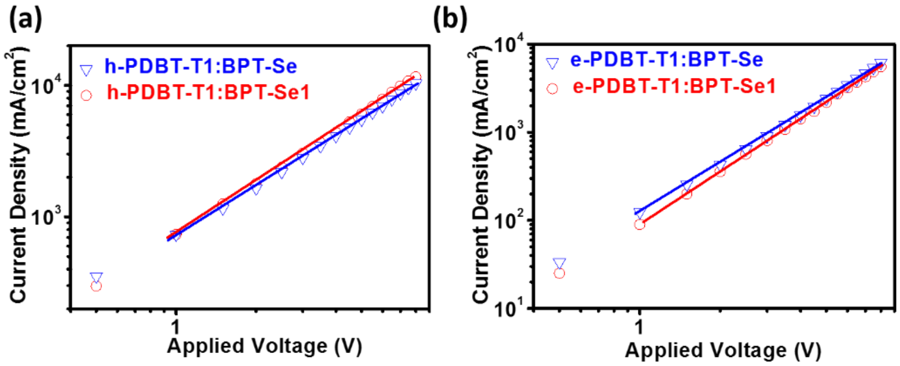

Figure S6. Space-charge-limited (SCLC) *J-V* characteristics of PDBT-T1/acceptors under dark condition, a) Electron-Only and b) Hole-Only

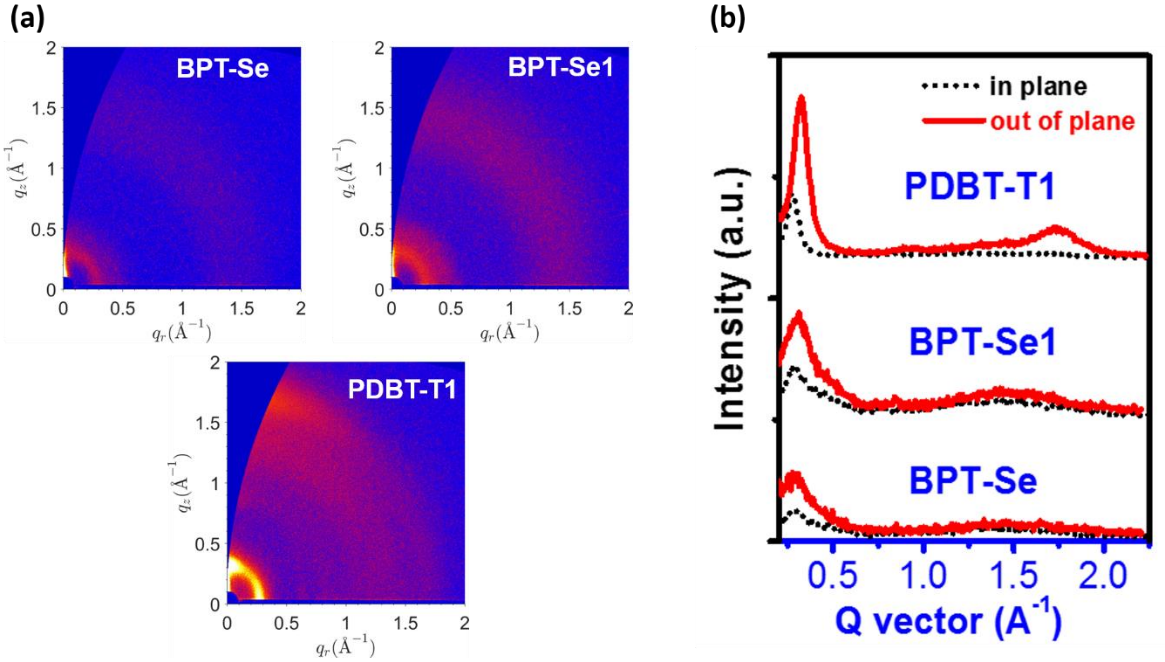

Figure S7. (a) 2D GIWAXS patterns of pure BPT-Se, BPT-Se1 and PDBT-T1 films; (b) The corresponding intensity profiles along the in-plane (black line) and out-of-plane (red line) of BPT-Se, BPT-Se1 and PDBT-T1 films.

Figure S8. ^{1}H NMR spectrum of compound **3**.

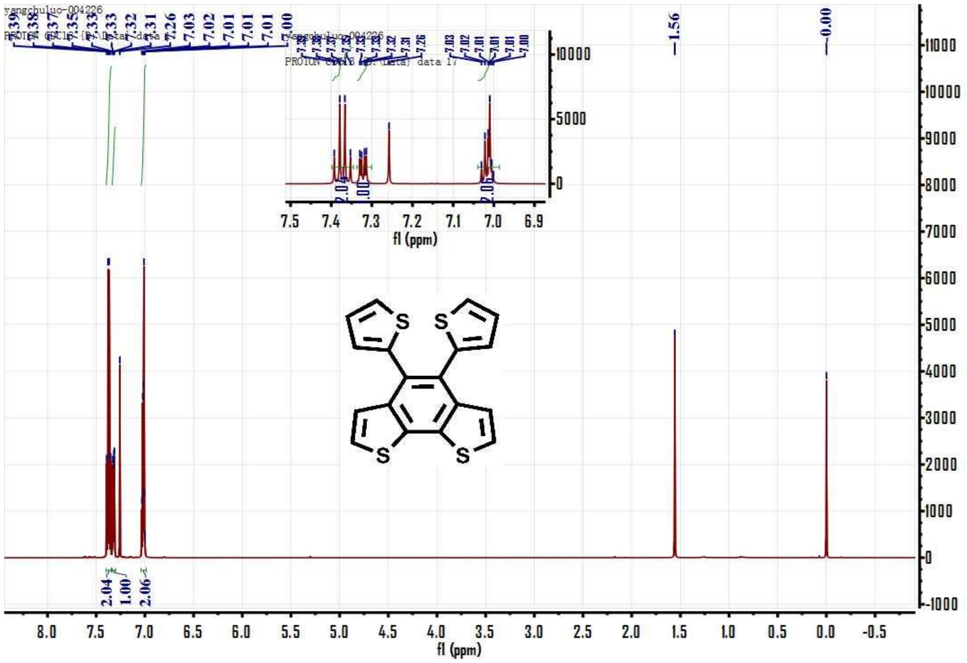

Figure S9. ^{1}H NMR spectrum of compound **4**.

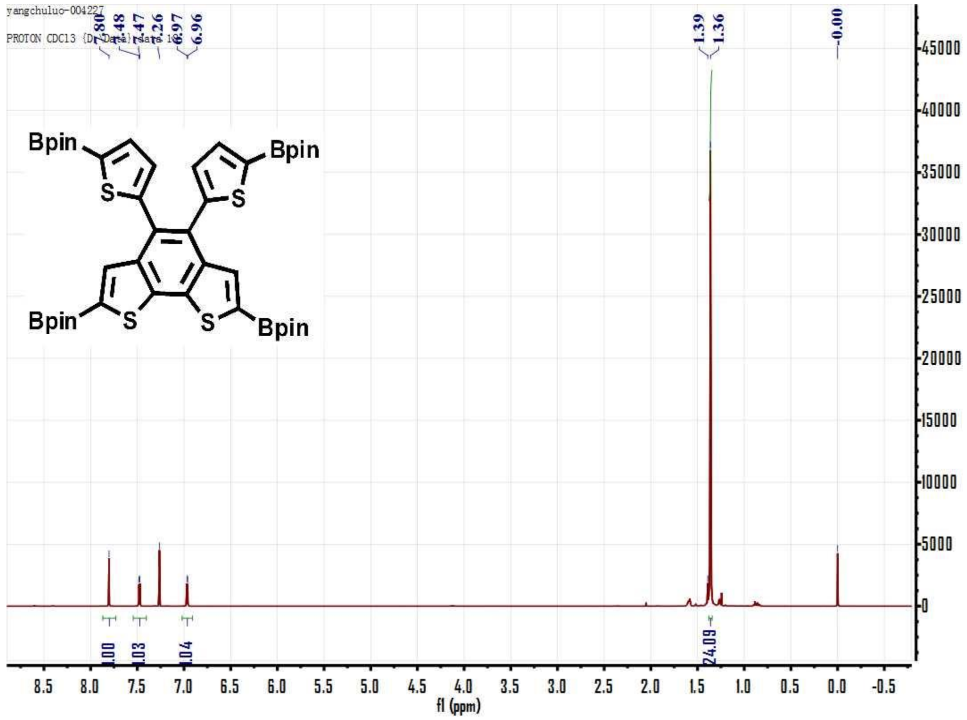

Figure S10. ^{1}H NMR spectrum of BPT-Se.

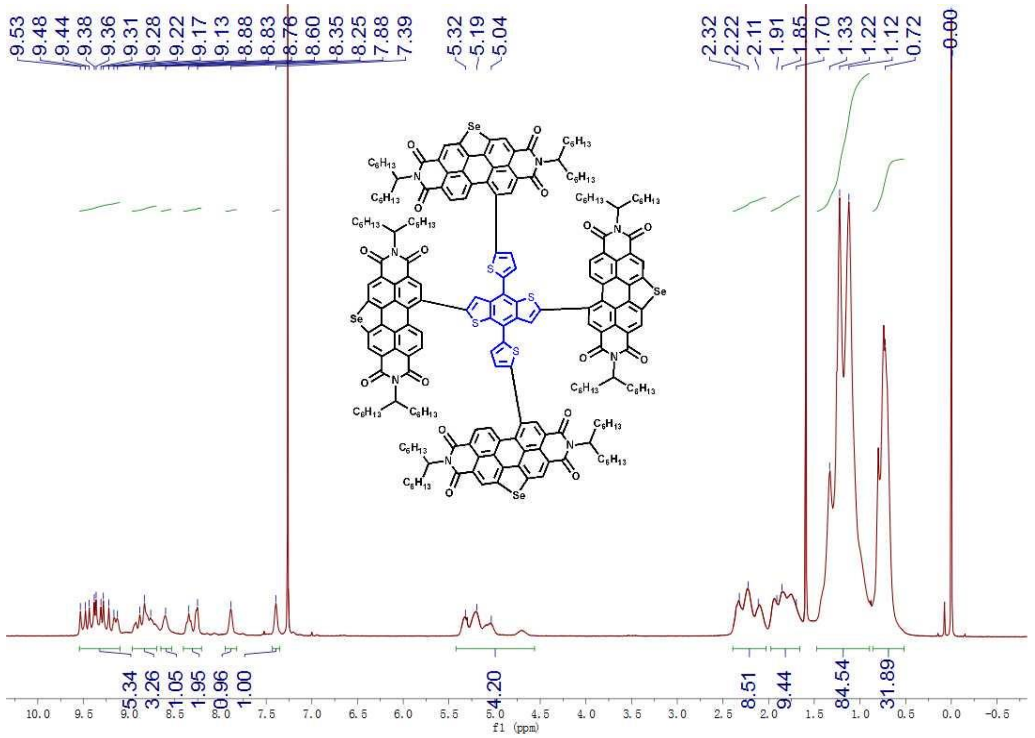

Figure S11. ^{1}H NMR spectrum of BPT-Se1.

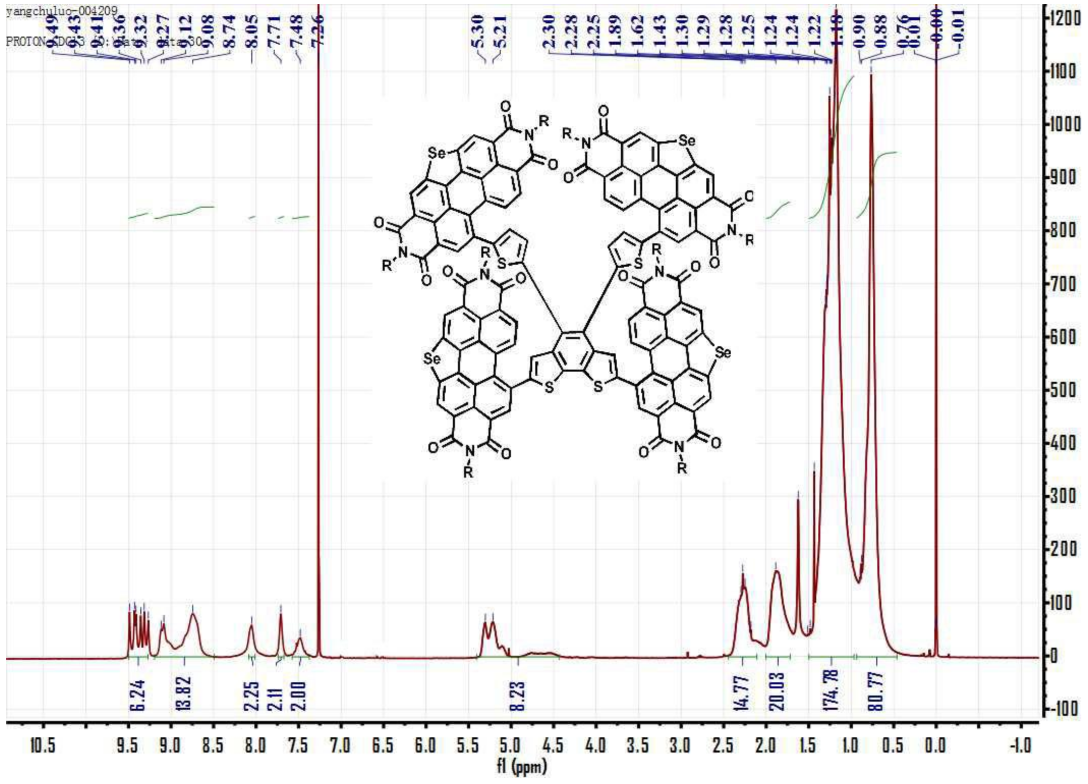

References

1. M. J. Frisch, G. W. Trucks, H. B. Schlegel, G. E. Scuseria, M. A. Robb, J. R. Cheeseman, G. Scalmani, V. Barone, B. Mennucci, G. A. Petersson, H. Nakatsuji, M. Caricato, X. Li, H. P. Hratchian, A. F. Izmaylov, J. Bloino, G. Zheng, J. L. Sonnenberg, M. Hada, M. Ehara, K. Toyota, R. Fukuda, J. Hasegawa, M. Ishida, T. Nakajima, Y. Honda, O. Kitao, H. Nakai, T. Vreven, J. A. Montgomery Jr., J. E. Peralta, F. Ogliaro, M. Bearpark, J. J. Heyd, E. Brothers, K. N. Kudin, V. N. Staroverov, R. Kobayashi, J. Normand, K. Raghavachari, A. Rendell, J. C. Burant, S. S. Iyengar, J. Tomasi, M. Cossi, N. Rega, J. M. Millam, M. Klene, J. E. Knox, J. B. Cross, V. Bakken, C. Adamo, J. Jaramillo, R. Gomperts, R. E. Stratmann, O. Yazyev, A. J. Austin, R. Cammi, C. Pomelli, J. W. Ochterski, R. L. Martin, K. Morokuma, V. G. Zakrzewski, G. A. Voth, P. Salvador, J. J. Dannenberg , S. Dapprich , A. D. Daniels , O. Farkas ,J. B. Foresman , J. V. Ortiz , J. Cioslowski, D. J. Fox, Gaussian, Inc., Wallingford CT, **2009**.
2. L. Goerigk, A. Hansen, C. Bauer, S. Ehrlich, A. Najibi and S. Grimme, *Phys. Chem. Chem. Phys.* **2017**, *19*, 32184.
3. T. Harschneck, N. Zhou, E. F. Manley, S. J. Lou, X. Yu, M. R. Butler, A. Timalsina, R. Turrisi, M. A. Ratner, L. X. Chen, R. P. Chang, A. Facchetti and T. J. Marks, *Chem. Commun.* **2014**, *50*, 4099-4101.
4. Z. Luo, T. Liu, W. Cheng, K. Wu, D. Xie, L. Huo, Y. Sun and C. Yang, *J. Mater. Chem. C* **2018**, *6*, 1136-1142.
5. J. M. Villar, J. Suarez, J. A. Varela and C. Saa, *Org. Lett.* **2017**, *19*, 1702-1705.
6. Z. Luo, T. Liu, W. Cheng, K. Wu, D. Xie, L. Huo, Y. Sun, C. Yang, *J. Mater. Chem. C* **2018**, *6*, 1136-1142.

7. T. Liu, D. Meng, Y. Cai, X. Sun, Y. Li, L. Huo, F. Liu, Z. Wang, T. P. Russell, Y. Sun, *Adv. Sci.* **2016**, *3*, 1600117.
8. D. Meng, D. Sun, C. Zhong, T. Liu, B. Fan, L. Huo, Y. Li, W. Jiang, H. Choi, T. Kim, J. Y. Kim, Y. Sun, Z. Wang, A. J. Heeger, *J. Am. Chem. Soc.* **2016**, *138*, 375.
9. Q. Wu, D. Zhao, A. M. Schneider, W. Chen, L. Yu, *J. Am. Chem. Soc.* **2016**, *138*, 7248.
10. D. Meng, H. Fu, C. Xiao, X. Meng, T. Winands, W. Ma, W. Wei, B. Fan, L. Huo, N. L. Doltsinis, *J. Am. Chem. Soc.* **2016**, *138*, 10184.
11. Q. Wu, D. Zhao, J. Yang, V. Sharapov, Z. Cai, L. Li, N. Zhang, A. Neshchadin, W. Chen, L. Yu, *Chem. Mater.* **2017**, *29*, 1127.
12. A. D. Hendsbee, J.-P. Sun, W. K. Law, H. Yan, I. G. Hill, D. M. Spasyuk, G. C. Welch, *Chem. Mater.* **2016**, *28*, 7098.
13. Y. Duan, X. Xu, H. Yan, W. Wu, Z. Li, Q. Peng, *Adv. Mater.* **2017**, *29*, 1605115.
14. N. Liang, K. Sun, Z. Zheng, H. Yao, G. Gao, X. Meng, Z. Wang, W. Ma, J. Hou, *Adv. Energy Mater.* **2016**, *6*, 1600060.
15. S. Li, W. Liu, C.-Z. Li, T.-K. Lau, X. Lu, M. Shi, H. Chen, *J. Mater. Chem. A* **2016**, *4*, 14983.
16. J. Liu, S. Chen, D. Qian, B. Gautam, G. Yang, J. Zhao, J. Bergqvist, F. Zhang, W. Ma, H. Ade, O. Inganäs, K. Gundogdu, F. Gao, H. Yan, *Nat. Energy* **2016**, *1*, 16089.
17. A. Zhang, C. Li, F. Yang, J. Zhang, Z. Wang, Z. Wei and W. Li, *Angew. Chem. Int. Ed.* **2017**, *56*, 2694-2698.

18. J. Zhang, Y. Li, J. Huang, H.Hu, G. Zhang, T. Ma, P. C.Y. Chow, H. Ade, D. Pan, and H. Yan, *J. Am. Chem. Soc.* **2017**, *139*, 16092.
